# C-Type Lectin Receptors in Host Defense Against Bacterial Pathogens

**DOI:** 10.3389/fcimb.2020.00309

**Published:** 2020-07-07

**Authors:** Malgorzata E. Mnich, Rob van Dalen, Nina M. van Sorge

**Affiliations:** ^1^Medical Microbiology, UMC Utrecht, Utrecht University, Utrecht, Netherlands; ^2^GSK, Siena, Italy; ^3^Interfaculty Institute of Microbiology and Infection Medicine, University of Tübingen, Tübingen, Germany; ^4^Department of Medical Microbiology and Infection Prevention, Amsterdam University Medical Center, University of Amsterdam, Amsterdam, Netherlands; ^5^Netherlands Reference Laboratory for Bacterial Meningitis, Amsterdam University Medical Center, Amsterdam, Netherlands

**Keywords:** bacteria, antigen-presenting cells, immunity, glycan, host-pathogen interaction, C-type lectin, pattern-recognition receptor

## Abstract

Antigen-presenting cells (APCs) are present throughout the human body—in tissues, at barrier sites and in the circulation. They are critical for processing external signals to instruct both local and systemic responses toward immune tolerance or immune defense. APCs express an extensive repertoire of pattern-recognition receptors (PRRs) to detect and transduce these signals. C-type lectin receptors (CLRs) comprise a subfamily of PRRs dedicated to sensing glycans, including those expressed by commensal and pathogenic bacteria. This review summarizes recent findings on the recognition of and responses to bacteria by membrane-expressed CLRs on different APC subsets, which are discussed according to the primary site of infection. Many CLR-bacterial interactions promote bacterial clearance, whereas other interactions are exploited by bacteria to enhance their pathogenic potential. The discrimination between protective and virulence-enhancing interactions is essential to understand which interactions to target with new prophylactic or treatment strategies. CLRs are also densely concentrated at APC dendrites that sample the environment across intact barrier sites. This suggests an–as yet–underappreciated role for CLR-mediated recognition of microbiota-produced glycans in maintaining tolerance at barrier sites. In addition to providing a concise overview of identified CLR-bacteria interactions, we discuss the main challenges and potential solutions for the identification of new CLR-bacterial interactions, including those with commensal bacteria, and for in-depth structure-function studies on CLR-bacterial glycan interactions. Finally, we highlight the necessity for more relevant tissue-specific *in vitro, in vivo* and *ex vivo* models to develop therapeutic applications in this area.

## Introduction

A large variety of bacterial species, collectively called the microbiome, lives in and on the human body. Most of these species have beneficial effects on human health, but opportunistic pathogens are also frequently present. In case of barrier defects or (temporary) immune system impairments, these microbes can enter tissues with the risk of causing local or, upon further dissemination, systemic infections. Fortunately, the human immune system is well-equipped with a wide range of innate pattern-recognition receptors (PRRs) that sense specific constituents or microbe-associated molecular patterns of bacteria. Among these PRRs, four different families are currently distinguished (Takeuchi and Akira, 2010). Nucleotide-binding oligomerization domain-like receptors and retinoic acid-inducible gene-I-like receptors (RLRs) are the cytoplasmic sensors of a cell, whereas Toll-like receptors (TLRs) are expressed in endosomes and on the cell surface (Takeuchi and Akira, 2010). The family of C-type lectin receptors (CLRs) include both transmembrane and soluble extracellular proteins (Brown et al., 2018). On tissue-resident antigen-presenting cells (APCs), engagement of PRRs including CLRs triggers local cytokine production, which is important to attract other immune cells such as neutrophils from the circulation to the site of infection and clear invading microbes (Janela et al., 2019; Sparber et al., 2019). Moreover, CLR-induced APC activation is instrumental to instruct adaptive immunity, including T cell polarization through production of cytokines and expression of co-stimulatory molecules, antibody production as well as immunological memory formation (Brown et al., 2018). The induction of these adaptive responses is indispensable for protection from reinfection and key for efficacy of vaccines. Therefore, a better understanding of CLR-bacterial glycan interactions could aid the development of therapeutic applications and vaccines.

CLRs are specialized in recognition of exposed sugar residues or sugar motifs present on self as well as non-self structures (Hoving et al., 2014; Yan et al., 2015). Recognition of specific glycans occurs through one or more carbohydrate recognition domains (CRDs) in a Ca^2+^-dependent manner. CLRs expressed on APCs can be divided based on their topology as type I and type II transmembrane proteins; type I receptors are characterized by the N-terminus pointing out of the cell and multiple CRDs, whereas type II receptors have their N-terminus directed toward the cytoplasm and an extracellular C-terminus that contains a single CRD (van Kooyk, 2008). Receptors of both groups have a stalk region, a transmembrane region and an intracellular domain with or without a signaling motif. Within type I and type II receptors, CLRs can additionally be categorized based on conserved amino acid motifs in their CRDs that determine their glycan specificity and Ca^2+^ coordination. CLRs with an EPN (Glu-Pro-Asn) amino acid motif in their CRD, such as DC-SIGN (CD209), langerin (CD207) and mannose receptor (MR, CD206), preferentially bind glycans with equatorial 3- and 4-hydroxyl groups such as mannose, fucose, and *N*-acetylglucosamine (GlcNAc) residues. On the other hand, CLRs with a QPD (Gln-Pro-Asp) motif preferentially bind glycans with axial 4-hydroxyl groups such as galactose and *N*-acetylgalactosamine (GalNAc) terminated glycans (Drickamer, 1992). In humans, most CLRs possess the EPN motif, with the exception of Macrophage Galactose-type C-type lectin (MGL, CD301), which possesses the QPD motif (Drickamer and Taylor, 2015). Despite the shared structural features of their CRDs, CLRs display considerable variation in overall structure, cellular expression profiles and signal transduction cascades. These differences have important consequences for the specific contributions of CLRs to antimicrobial immunity, since they strongly affect ligand specificity and the induced immune responses, which can either support host defense or allow immune escape. The role of CLRs in host defense against fungal, viral and mycobacterial infections has recently been reviewed by others (Liu et al., 2017; Shiokawa et al., 2017; Bermejo-Jambrina et al., 2018). Instead, our review focuses on recent findings on the importance of CLRs in recognition of and responses to bacterial pathogens by APCs. In addition, we highlight tools and technologies used for the identification of new interactions, and discuss challenges in the choice of appropriate cell model systems and in the translation of *in vitro* to *in vivo* studies. We conclude our review with possible applications of the gathered knowledge for the development of new CLR targeting strategies in vaccines or CLR blocking to counter bacterial immune evasion (Lang et al., 2011; Wamhoff et al., 2019).

## Recognition of Bacterial Glycans by Tissue-Resident APCs

The bacterial cell wall is essential for bacterial survival; it defines bacterial cell shape, is critical to sequester ions for bacterial homeostasis and serves as a scaffold for proteins and glycopolymers, to name but a few important features (Silhavy et al., 2010; Dorr et al., 2019). As such, it is much more than a structure that provides resistance to physical stress or harmful environmental factors. In fact, the bacterial cell wall and all its associated structures provide an important interface for direct sensing and communication with the environment, including the host. Despite considerable differences in overall cell wall composition between Gram-positive and -negative bacteria, both classes of bacteria decorate their cell wall with glycans. The best studied examples are capsular polysaccharides, lipopolysaccharide (LPS) and peptidoglycan. Capsular polysaccharides and LPS are effective vaccine antigens when conjugated to protein carriers (glycoconjugate vaccines), whereas proteins in the peptidoglycan biosynthesis pathways are proven targets of antibiotics (Schneider and Sahl, 2010; Rappuoli, 2018). However, bacteria produce a much broader array of glycan structures, which are incorporated in glycolipids, proteins, flagella and glycopolymers (Tytgat and Lebeer, 2014). All these structures are potential ligands for CLRs, and considerable insight into specific molecular interactions has been made the past decades (Prado Acosta and Lepenies, 2019). Importantly, these studies have revealed that interactions between bacterial glycans and CLRs do not always support host defense. Instead, bacteria can exploit CLR interactions for immune evasion, resulting in subversion of host defense responses and increased morbidity. Consequently, detailed molecular and functional insight into bacterial glycan recognition by CLRs is critical to distinguish beneficial from detrimental interactions and inform the development of new treatment or prophylactic strategies.

**Figure 1:**
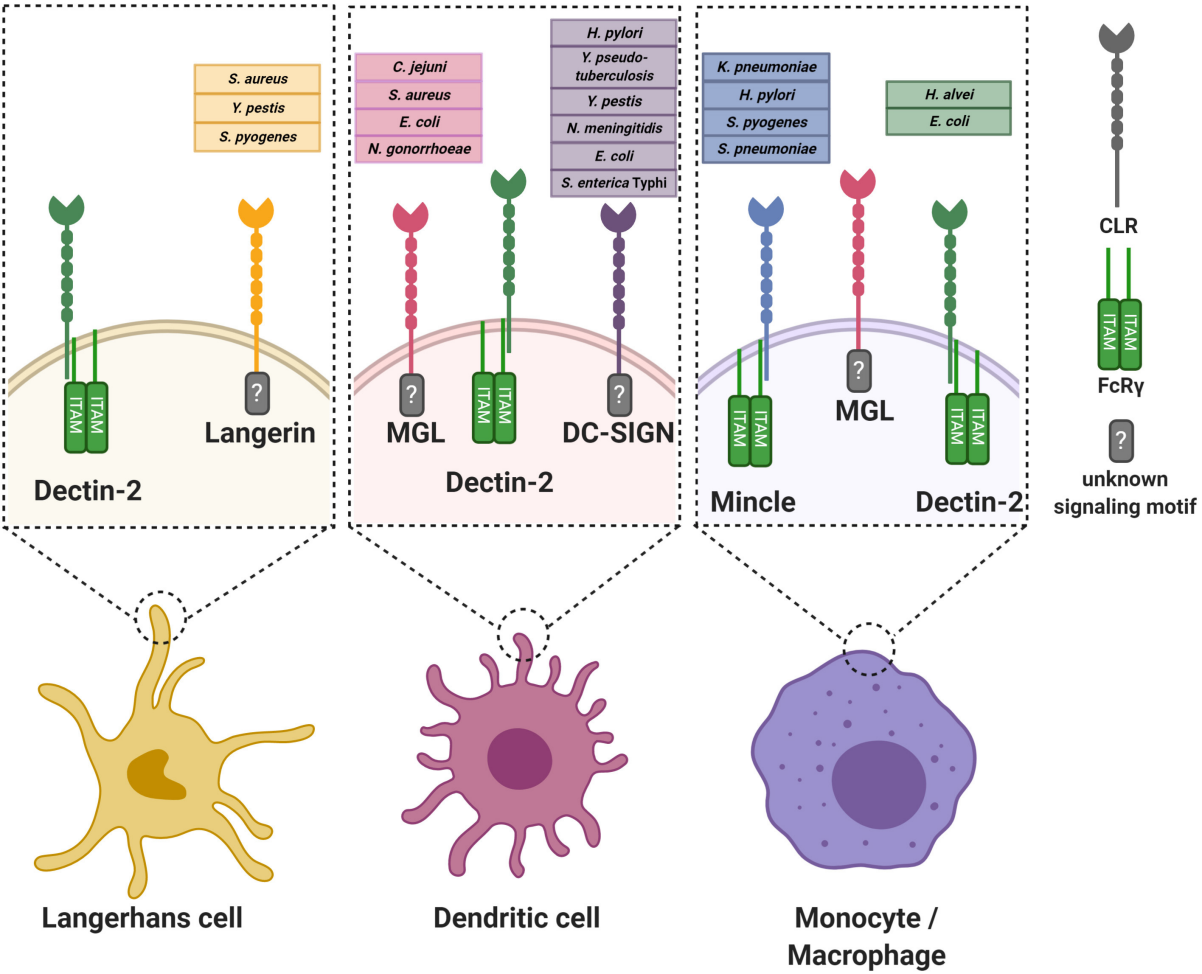
Graphical overview of the discussed C-type lectin receptors on various antigen-presenting cells. For every receptor, the known interacting bacterial species are indicated. Where known, the intracellular signaling motif or associated signaling adaptor molecule is stated. For simplicity, the oligomerization state of the CLRs is not incorporated in the Figure. FcRγ, Fc receptor gamma chain.

The functional consequences of CLR engagement are determined by the encountered bacterial ligands but also on the location in the body where the interaction occurs. First, different tissues are populated with specific APC subtypes, which can be phenotypically distinguished from each other by presence of specific immunological markers (Bigley et al., 2015; Alculumbre and Pattarini, 2016; Gunawan et al., 2016; Alcantara-Hernandez et al., 2017; Collin and Bigley, 2018). Second, the local microenvironment provides specific signals to induce APC tissue adaptation, resulting in different receptor expression profiles and migratory capacities of similar APC subtypes in different tissues (Lundberg et al., 2013; Alcantara-Hernandez et al., 2017; Collin and Bigley, 2018). Here, we summarize and discuss specific interactions between CLRs and bacterial glycans (Figure 1), categorized by tissue as relevant for site of bacterial entry.

### Skin

The skin represents the largest organ of the body and is colonized by a plethora of microorganisms (Byrd et al., 2018). Immune cells of the skin constantly interact with microbes and their products, even deeper within the tissue, without causing infection or inflammation (Nakatsuji et al., 2013). However, two common skin resident species, *Staphylococcus aureus* and *Streptococcus pyogenes*, are also frequent causes of skin infections (Cardona and Wilson, 2015). Skin infections caused by these pathogens are diverse in their presentation and pose a risk for life-threatening systemic infections. Both these skin-tropic pathogens, but not other more abundant species, such as *Staphylococcus epidermidis* and *Staphylococcus lugdunensis*, interact with the CLR langerin (CD207) as confirmed by experiments using recombinant human langerin as well as ectopically-expressing langerin cell lines (van Dalen et al., 2019a,b). Langerin is exclusively expressed on human Langerhans cells (LCs), which are a subclass of myeloid cells that are abundantly present in the skin epidermis. LCs form an important first line of defense against pathogens (Doebel et al., 2017; Deckers et al., 2018). Although most studies focus on skin LCs, their localization is not restricted to the skin; they are also present in mucosal tissues and other epithelial linings, for example foreskin, cervical mucosal tissue, tonsils, tongue and the upper respiratory tract (Patterson et al., 2002; van der Vlist et al., 2011).

Langerin is expressed as a trimer through oligomerization of the neck region, and its cytoplasmic tail contains a putative proline-rich signaling motif (Valladeau et al., 2000; Stambach and Taylor, 2003; Tateno et al., 2010) (Table 1). Based on binding profiles of recombinant human langerin to individual microbes, glycan arrays, langerin has specificity for sulfated and mannosylated glycans as well as β-glucans, which it binds in a Ca^2+^-dependent manner (de Jong et al., 2010b; Feinberg et al., 2010, 2011; Hanske et al., 2017). However, these particular glycans are unlikely to be involved in the interaction with *S. pyogenes* or *S. aureus*, as they are not produced by these bacterial species. Although the ligand of langerin on *S. pyogenes* has not been identified yet (van Dalen et al., 2019b), human pharyngeal LCs have been observed to interact with *S. pyogenes* (Reed et al., 1994). On *S. aureus*, the langerin ligand depends on the gene *tarS* as confirmed by binding studies comparing wild-type and isogenic mutant strains (van Dalen et al., 2019a). *TarS* encodes a glycosyltransferase that attaches a conserved β1,4-linked *N*-acetylglucosamine (β1,4-GlcNAc) moiety on the surface glycopolymer wall teichoic acid (WTA) (Brown et al., 2012), which is an important structural component of the Gram-positive bacterial cell wall (Brown et al., 2013). Recognition of WTA β1,4-GlcNAc increases cytokine production of Th1- and Th17-skewing cytokines by *in vitro*-generated LCs. Correspondingly, epicutaneous infection with *S. aureus* producing β1,4-GlcNAc increases skin inflammation in human langerin transgenic mice (van Dalen et al., 2019a). The use of langerin transgenic mice was required since the murine langerin homolog does not recognize *S. aureus* (van Dalen et al., 2019a), which is of interest as *S. aureus* is not a mouse commensal. Species-specificity for this interaction may therefore reflect the extended co-evolution of *S. aureus* with the human host. Approximately 30% of the *S. aureus* strains are able to co-decorate WTA with the accessory modification α1,4-GlcNAc, which requires the glycosyltransferase TarM (Xia et al., 2010). Langerin does not interact with α1,4-GlcNAc-modified WTA, but co-expression of α1,4-GlcNAc and β-GlcNAc decreased interaction with langerin, suggestion a possible strategy to evade detection by langerin (van Dalen et al., 2019a) Recently, a third WTA glycosyltransferase, TarP, was identified in 5–10% of *S. aureus* strains (Gerlach et al., 2018). TarP attaches β1,3-GlcNAc to WTA (Gerlach et al., 2018), but it is currently unknown whether or how this modification impacts interaction with langerin or responses by LCs. Several SNPs in the langerin CRD alter the ligand specificity of langerin (Feinberg et al., 2013). These SNPs impair *S. aureus* recognition and uptake by langerin-expressing cells, thereby providing a potential increase in disease susceptibility (van Dalen et al., 2019b). Intriguingly, interaction with *S. pyogenes* was much less affected by these SNPs. Although preliminary, these data seem to suggest that such genetic variation does not affect overall susceptibility to bacterial infections (van Dalen et al., 2019b).

**Table 1:** Overview of human CLRs and their characteristics discussed in this review, and their characteristics.

**Common names**	**Cellular expression pattern**	**Signaling**	**Glycan specificity**	**Recognized bacteria**
Langerin (CD207, CLEC4K)	LCs (Valladeau et al., 2000)	Unknown but contains intracellular proline-rich W**P**RE**P**P motif (Thépaut et al., 2009)	Mannose, fucose, GlcNAc (Feinberg et al., 2011)	*S. aureus* (van Dalen et al., 2019a), *S. pyogenes* (van Dalen et al., 2019b), *Y. pestis* (Yang et al., 2015)
MGL (CD301, CLEC10A)	Macrophages, DCs in skin and lymphoid organs (Raes et al., 2005; van Vliet et al., 2008).	Unknown	Galactose, α- and β-GalNAc (Suzuki et al., 1996)	*S. aureus* ST395 (Mnich et al., 2019), *C. jejuni* (van Sorge et al., 2009), *N. gonorrhoeae* (van Vliet et al., 2009), *E. coli* (Maalej et al., 2019)
DC-SIGN (CD209, CLEC4L)	DCs at mucosal surfaces, skin dermis and lymphoid organs, macrophages (Geijtenbeek et al., 2000a,b; Soilleux et al., 2002).	Unknown	Mannose, α1-3 and α1-4 fucosylated glycans, GlcNAc (Suzuki et al., 1996; van Vliet et al., 2005)	*H. pylori* (Bergman et al., 2004), *Y. pseudotuberculosis* (Zhang et al., 2006b), *Y. pestis* (Zhang et al., 2008)*, E. coli* K12 (Zhang et al., 2006b), *N. meningitidis* (Steeghs et al., 2006), *N. gonorrhoeae* (Zhang et al., 2006a), *Salmonella enterica* serovar Typhimurium (Zhang et al., 2006b)
Mincle (CLEC4E)	Activated macrophages, some subpopulations of B cells (Matsumoto et al., 1999; Kawata et al., 2012)	ITAM motif in associated FcRγ chain (Matsumoto et al., 1999; Yamasaki et al., 2008)	Broad range of self and non-self glycolipids (Lu et al., 2018)	*K. pneumoniae* (Sharma et al., 2014), *H. pylori* (Devi et al., 2015)*, S. pyogenes* (Imai et al., 2018)*, S. pneumoniae* (Imai et al., 2018)
Dectin-2 (CLEC6A)	Macrophages, DCs, LCs, monocytes (Ariizumi et al., 2000; Taylor et al., 2005)	ITAM motif in associated FcRγ chain (Sato et al., 2006)	α-mannans (Fernandes et al., 1999; Sato et al., 2006; Saijo et al., 2010)	*Hafnia alvei* (Wittmann et al., 2016)*, E. coli* O9a (Wittmann et al., 2016)

*DC, dendritic cell; LC, Langerhans cell, ITAM, intracellular tyrosine-based activation motif; GlcNAc, N-acetylglucosamine; GalNAc, N-acetylgalactosamine*.

Another bacterium that can enter the body through the skin is the Gram-negative pathogen *Yersinia pestis*, the cause of plague. Infections with *Y. pestis* can occur after a bite from an infected flee, which delivers the bacterium directly into the tissue, past the protective layer of stratum corneum. Through interaction with langerin, *Y. pestis* can be phagocytosed by LCs (Yang et al., 2015). In addition to langerin, *Y. pestis* also interacts with Dendritic-cell-specific intracellular adhesion molecule-3-grabbing non-integrin (DC-SIGN, CD209) (Zhang et al., 2008), which is expressed on dendritic cells (DCs), but not LCs, in mucosal linings, skin dermis, and lymphoid organs and macrophages (Geijtenbeek et al., 2000a,b; Soilleux et al., 2002). DC-SIGN is expressed as a tetramer on the cell surface, with each monomer consisting of a single CRD, neck region and an intracellular domain (van Kooyk and Geijtenbeek, 2003). Although triggering of DC-SIGN alone does not induce DC responses, activation of DC-SIGN modulates signaling cascades induced by other PRRs (Gringhuis et al., 2009). DC-SIGN selectively recognizes oligosaccharides containing high mannose residues but also fucosylated structures in a Ca^2+^-dependent manner and tetramerization of the receptor favors the recognition of closely-spaced mannose residues on the target molecule (Mitchell et al., 2001; Feinberg et al., 2005; Yu et al., 2009). For *Y. pestis*, the interaction with langerin and DC-SIGN occurs through the core oligosaccharide of LPS and strains that are genetically-engineered to cover the LPS core are defective in DC and LC invasion (Zhang et al., 2008; Yang et al., 2015). Lectin-dependent phagocytosis seems to favor dissemination of *Y. pestis* by facilitating migration of bacteria to the lymph nodes (Yang et al., 2015, 2019). However, additional infection experiments in knock-out mice are required to specifically link the observed phenotype to the langerin or DC-SIGN mouse homolog (Zhang et al., 2008; Yang et al., 2015, 2019).

Skin macrophages and DCs, but not LCs, express the CLR Macrophage Galactose-type C-type lectin (MGL, CD301) (Raes et al., 2005; van Vliet et al., 2008). MGL is a homotrimeric receptor that interacts in a Ca^2+^-dependent manner with galactose and terminal GalNAc residues through the QPD motif in its CRD (Suzuki et al., 1996; Marcelo et al., 2014; Tanaka et al., 2017). Recently, a specific lineage of *S. aureus* (ST395) was shown to interact with MGL, as based on binding experiments with recombinant human MGL (Mnich et al., 2019). This interaction depends on the unique WTA structure of this *S. aureus* lineage, which is composed of a glycerol-phosphate (GroP) backbone (as opposed to ribitol-phosphate in all other described *S. aureus* lineages) decorated with α-*N*-acetylgalactosamine (α-GalNAc) (Winstel et al., 2013, 2014). WTA α-GalNAc expression by wild-type *S. aureus* confers binding to human monocyte-derived dendritic cells (moDCs) and induces proinflammatory cytokine production compared to S. *aureus* strains genetically modified to lack α-GalNAc expression (Mnich et al., 2019). It remains to be determined whether the observed responses correspond to the responses of primary human macrophages or DCs isolated from skin. This knowledge is highly relevant to our understanding of the skin immune detection of these *S. aureus* strains.

In addition to interaction with langerin, *S. pyogenes* cell wall components also activate Macrophage inducible C-type lectin (Mincle). Mincle is modestly expressed under homeostatic conditions but significantly upregulated on resident and attracted myeloid cells and neutrophils upon infection or sterile inflammation in murine and human skin (Iborra et al., 2016; Kostarnoy et al., 2017). Increased expression results from increased transcriptional activity induced by inflammatory cytokines such as TNF-α, IL-6, and IFN-γ (Matsumoto et al., 1999). Enhanced surface expression and phagocytic capacity is also conferred by the formation of heteromeric complexes of Mincle with other CLRs such as macrophage C-type lectin (MCL) (Lobato-Pascual et al., 2013). Despite the presence of an EPN motif in its CRD, which usually confers specificity for mannan, all Mincle ligands identified so far contain a lipid moiety in addition to the glycan, suggesting that glycolipids are the molecular signature required for Mincle recognition (Lu et al., 2018). Mincle induces cellular activation through coupling with the Fc receptor common γ chain (FcRγ), which contains an intracellular ITAM signaling motif (Matsumoto et al., 1999; Yamasaki et al., 2008). Mincle interacts with glycolipid antigens from *S. pyogenes*, specifically the lipophilic components monoglucosyldiacylglycerol (MGDG) and diglycosyldiacylglycerol (DGDG) (Imai et al., 2018), which constitute membrane anchors for lipoteichoic acid (LTA) in the bacterial cell envelope. Intriguingly, MGDG activates DCs via Mincle, resulting in antigen-induced IL-17 production from CD4+ T cells, whereas DGDG prevents MGDG-induced cellular activation through the same receptor (Imai et al., 2018). Abrogation of Mincle-mediated *S. pyogenes* detection impairs production of pro-inflammatory cytokines, resulting in increased bacteremia and mortality in a mouse model of systemic infection (Imai et al., 2018). Mincle-mediated protection depends on induction of interferon-γ by a specialized lineage of immature myeloid cells, which in turn requires TLR2-induced IL-6 production by these same cells (Matsumura et al., 2019). As MGDG and DGDG are a product of the same biosynthetic pathway, controlled expression of these different lipid anchors may allow *S. pyogenes* to escape Mincle detection.

Overall, these examples highlight that APC subsets in the skin express distinct CLR repertoires that affect the molecular interaction and immunological responses upon bacterial infection. As DCs, macrophages and LCs are located at different anatomical depths, it is likely that the depth of infection is an important determinant for the outcome of infection. We believe this represents an important area of research to understand pathogenesis of bacterial skin infections (Quaresma, 2019).

### Genital Tract

DCs and LCs are both present at barrier sites of the genital tract. *Neisseria gonorrhoeae* is a Gram-negative pathogenic bacterium and the cause of the sexually-transmitted disease gonorrhea. It can express different lipooligosaccharide (LOS) variants that correlate with altered disease states, i.e. active disease is associated with *N. gonorrhoeae* variant C (Schneider et al., 1991). *N. gonorrhoeae* variants A, B, and C only differ in their LOS glycosylation, expressing terminal GlcNAc, galactose and GalNAc, respectively (van Vliet et al., 2009). Using these three well-defined *N. gonorrhoeae* variants, recombinant soluble CLR-constructs and human moDCs, it was discovered that *N. gonorrhoeae* variant C interacts with MGL, whereas *N. gonorrhoeae* variant A is detected by DC-SIGN. Engagement of MGL on moDCs by *N. gonorrhoeae* variant C shifts DC cytokine secretion and subsequent T helper differentiation toward Th2 in a co-culture system compared to *N. gonorrhoeae* variants A and B (Zhang et al., 2006a; van Vliet et al., 2009). The interaction with MGL is thought to benefit bacterial survival as a shift toward Th2 is considered to be a non-protective response against *N. gonorrhoeae* infection. So far, no interactions of LCs with bacteria relevant to genital tract infections or sexually-transmitted diseases have been studied.

### Gastrointestinal Tract

The stomach is a hostile environment due to its very low pH. In fact, it was assumed for a long time that this was a sterile site of the body. However, *Helicobacter pylori*, a Gram-negative bacterium and cause of ulcers and stomach cancer, has evolved unique strategies to survive and colonize the gastric mucosa. Intriguingly, *H. pylori* expresses phase-variable LPS and LPS variants containing Lewis epitopes interact with DC-SIGN (Bergman et al., 2004). Functional *in vitro* experiments using human moDCs and *H. pylori* strains with and without Lewis antigen epitopes on their LPS, demonstrated that engagement of DC-SIGN blocks Th1 development (Bergman et al., 2004). This response is assumed to contribute to long-lasting colonization of *H. pylori* in a large proportion of the population. In addition to DC-SIGN, *H. pylori* also affects immune responses through Mincle as based on experiments with PMA-differentiated THP-1 cells, in which Mincle expression was strongly upregulated after exposure to *H. pylori*. Furthermore, Mincle expression decreases production of pro-inflammatory cytokines while concordantly increasing the production of anti-inflammatory IL-10 in response to *H. pylori* (Devi et al., 2015). Computational modeling studies indicate that Mincle could interact with LPS of *H. pylori* containing Lewis-antigens (Devi et al., 2015), but additional studies are required to confirm the interaction and relevance of *H. pylori*-Mincle interaction at a molecular level.

In contrast to the stomach, the intestines are colonized by a wealth of different bacterial species. Multiple interactions between pathogenic enteric bacteria and CLRs have been discovered. For example, DC-SIGN interacts with the core LPS, especially the GlcNAc residues, of many Gram-negative enteric pathogens, including *Salmonella enterica* serovar Typhimurium, *E. coli* K12 and *Yersinia pseudotuberculosis* (Zhang et al., 2006b). The interaction between *Y. pseudotuberculosis* and DC-SIGN promotes systemic dissemination and further infection in mice (He et al., 2019). For the other interactions the impact on infection is awaiting further studies. MGL is targeted by at least two gastrointestinal bacteria, *Campylobacter jejuni* and *E. coli* R1, through their LOS terminal GalNAc residues and, in case of *C. jejuni*, through expression of glycosylated proteins (van Sorge et al., 2009; Maalej et al., 2019). MGL targeting by *C. jejuni* suppresses IL-6 production by moDCs compared to isogenic *C. jejuni* mutant strains that lack *N*-glycan protein modifications (van Sorge et al., 2009). Finally, the CLR Dendritic-cell-associated C-type lectin-2 (Dectin-2/CLEC6A) interacts with mannosylated O-antigen of LPS from the nosocomial bacterial pathogens *Hafnia alvei* and *E. coli* O9a (Wittmann et al., 2016). Engagement of Dectin-2 by *H. alvei* mannosylated LPS enhances TLR4-induced cytokine responses of murine bone marrow-derived DCs (BM-DCs) as demonstrated using BM-DCs from Dectin-2 knockout mice (Wittmann et al., 2016). Dectin-2-induced signaling also synergizes with TLR4 responses in human myeloid cells and is dependent on Syk (Wittmann et al., 2016). These data suggest that Dectin-2 could have an important role in sensing bacteria that express mannosylated structures on their surface. The effect of Dectin-2 engagement for the outcome of infection requires further investigation.

### Respiratory Tract

The respiratory tract is continuously exposed to airborne microorganisms, including potential pathogens. The distribution and density of APC subsets differs along the respiratory tract. For example, LCs are much more abundant in the upper respiratory tract but are scarce when descending toward the lung (van der Vlist et al., 2011). On the other hand, subsets of DCs, which vary in expression of surface receptors and therefore likely in function, are observed within specific lung microenvironments (Patel and Metcalf, 2018). More precise and comprehensive information on CLR expression along the respiratory tract would be helpful to further interpret results from studies demonstrating that CLRs can protect from bacterial pneumoniae. Specifically, Mincle was shown to have a critical role in protection from pneumonia caused by *Klebsiella pneumoniae* and *Streptococcus pneumoniae*. For *K. pneumoniae*, no bacterial ligand was identified but the increased susceptibility to pneumonia is associated with an increased bacterial burden and exaggerated hyperinflammation (Sharma et al., 2014). The increased bacterial burden may be explained by defects in neutrophil-mediated clearance, as Mincle-deficient neutrophils show an impaired uptake of non-opsonized bacteria and impaired formation of neutrophil-extracellular traps (Sharma et al., 2014, 2017). A similar increase in pneumonia as well as mortality was observed in Mincle-knockout vs. wild-type mice challenged intrapulmonary with *S. pneumoniae* serotype 19F. The Mincle ligand of *S. pneumoniae* was identified as the LTA anchor moiety glucosyl-diacylglycerol (Glc-DAG), which is the same ligand as MGDG identified in *S. pyogenes* (Imai et al., 2018). Engagement of Mincle was not sufficient to protect mice from invasive pneumococcal disease as caused by *S. pneumoniae* serotype 3, which is more aggressive compared to serotype 19F as this strain causes bacteremia in addition to pneumonia (Behler-Janbeck et al., 2016). These results suggest that Mincle may contribute to local lung immunity against *S. pneumoniae* but is unable to contain bacteria once in the blood stream (Behler-Janbeck et al., 2016). However, it is very difficult to draw firm conclusions on the role of Mincle in pneumococcal infection, since the strains were not quantified for expression of the Mincle ligand. In addition, the two strains are genetically quite diverse, which likely alters the molecular context in which the Mincle ligand is expressed. As discussed above, this could lead to co-engagement of additional cellular receptors, leading to a different outcome of Mincle activation. Therefore, additional studies are required to fully unravel the role of Mincle in anti-pneumococcal lung immunity.

### Nasopharynx

*Neisseria meningitidis* can colonize the nasopharynx of healthy individuals but is also a common cause of invasive disease, most notably sepsis and meningitis. Dependent on the molecular composition of *N. meningitidis* LPS, the strain can interact with DC-SIGN with important consequences for uptake and induction of adaptive immunity. Truncation of *N. meningitidis* LPS (Δ*lgtB*; representative of immunotype L6) results in enhanced uptake by human moDCs through DC-SIGN and induction of anti-bacterial Th1 responses, as assessed by *in vitro* co-cultures with human primary cells (Steeghs et al., 2006). Immunotype L6 is rarely found among clinical isolates, suggesting that interaction with DC-SIGN is unfavorable for *N. meningitidis* survival (Steeghs et al., 2006). From an alternative perspective, immunotype L6 LPS may be used as a powerful vaccine adjuvant as it induces a favorable Th profile compared to unfavorable Th2 type responses with the currently approved human adjuvant alum.

### CLR-Mediated Transcompartmental Sampling

The studies described so far all involve interactions between CLRs and pathogenic or nosocomial bacterial species within tissues. However, interactions with commensal members of the microbiome are also likely to occur (Li T. H. et al., 2019). APCs at barrier sites such as the skin, gut or lung do not only respond to bacteria that have penetrated the epithelial barrier, but also have the capacity to actively sample the environment through extension of dendrites across the physical barriers (Rescigno et al., 2001; Niess et al., 2005; Jahnsen et al., 2006; Sung et al., 2006; Kubo et al., 2009; Thornton et al., 2012; Yoshida et al., 2014). CLRs are concentrated at the tips of APC protrusions and are able to trigger localized antigen uptake (Baranov et al., 2014), suggesting a critical role for CLRs in transcompartmental antigen sensing and uptake. The biological significance of this uptake process was demonstrated in a mouse model of staphylococcal scalded skin syndrome (SSSS), which depends on *S. aureus* exfoliating toxin (ET) (Ouchi et al., 2011). Patch-immunization induces specific cellular and humoral immunity that protects mice from SSSS which depends on the presence of LCs (Ouchi et al., 2011). Importantly, antigen-uptake through patch-immunization does not induce local inflammation or tissue damage (Ouchi et al., 2011). As such, non-invasive application is being explored for development of vaccines against infectious agents (Zheng et al., 2018). Investigations in this area may reveal a role for CLR-mediated APC transcompartmental uptake and activation in maintaining tissue homeostasis at barrier sites.

## The Impact of Biological Variation on CLR-Bacterial Interactions

In many of the studies described above, new interactions were identified using a representative bacterial strain of the species of interest and a single growth condition. Biologically-relevant interactions may be missed using such a limited set of experimental conditions. First, the bacterial target may only be expressed in a specific environment or under specific growth condition as a result of regulation. We have recently observed this for the interaction between MGL and *Staphylococcus lugdunensis* (Mnich et al., 2019). The gene encoding the bacterial glycosyltransferase TagN, responsible for the incorporation of GalNAc on WTA (Winstel et al., 2013, 2014), was identified in the *S. lugdunensis* genome, but no MGL binding to this bacterial strain was observed (Mnich et al., 2019). In contrast, constitutive expression of *S. lugdunensis tagN* from a plasmid did confer MGL binding (Mnich et al., 2019). These results seem to indicate lack of *tagN* expression in *S. lugdunensis* under our experimental growth conditions. Second, the target may only be present in a restricted subset or lineage from that specific species. Again, we can take the example of MGL interaction with GalNAc-decorated WTA, which is exclusive for the *S. aureus* ST395 lineage (Mnich et al., 2019). Overall, the glycan expression profile or repertoire on the bacterial surface after *in vitro* growth may not be representative for the *in vivo* situation. Variation between strains from a single species can be estimated based on genetic studies, but, as illustrated with the example of *S. lugdunensis* above, the presence of a gene does not guarantee expression under the used experimental conditions. In addition to genetic sequences, information on conditions that induce the gene or glycan biosynthesis cluster of interested can be estimated by transcriptional data from a wide range of growth conditions (Mader et al., 2016).

Even when a CLR-bacterial interaction is identified *in vitro*, translation to mouse models is often challenging as no murine homologs exist (e.g., DC-SIGN) or have vastly different glycan-binding profiles compared to the human CLR homolog (Hanske et al., 2016; van Dalen et al., 2019a). As an example of the latter, comparison of the glycan specificity using a bacterial glycan array for recombinantly-expressed murine and human langerin revealed a different binding pattern, in which murine langerin recognizes a broad set of bacterial glycans, but the binding pattern of human langerin is much more restricted (Hanske et al., 2017). Therefore, results from mouse infection models where CLRs are implicated should be interpreted with care with regard to translation of the results for human infection.

## Multivalency and Epitope Density in CLR-Ligand Interactions

CLRs not only differ from many other receptors due to their specificity for glycans, but also in their preference for binding multivalent ligands and densely-expressed epitopes (Dam and Brewer, 2010). Indeed, carbohydrate-protein interactions are usually of low affinity, which is overcome by multivalent display of the receptor and/or the ligand (Lepenies et al., 2013). In addition, multivalency and epitope density contribute to discrimination between self and non-self and are not just based on the identity of the glycan, since similar glycan epitopes are often found in pathogens and the host (Medzhitov and Janeway, 2002; Dam and Brewer, 2010). Indeed, data from sugar competition assays for several receptors revealed that glycans carrying multiple carbohydrate epitopes have higher ability to bind to these receptors than monosaccharides (Hsu et al., 2009). Studies that have systematically probed the effect of multivalency and configuration on binding capacity and cellular activation have been conducted for DC-SIGN and MGL (Eriksson et al., 2014; Li R. E. et al., 2019). Overall, higher glycan density increases lectin affinity and kinetics of binding, which is likely explained by one of the following concepts. The first is known as the ‘binding and sliding' mechanism, in which lectins constantly bind to and dissociate from the glycan epitope (Dam and Brewer, 2008). By increasing the number of glycan epitopes along the surface, the lectin-epitope complex becomes longer and of higher affinity. This theory can be applied to soluble lectins such as galectins (Dam and Brewer, 2008). In the second concept, lectins containing multiple CRDs align to allow simultaneous binding of multiple epitopes on a multivalent ligand in a face-to-face interaction (Dam and Brewer, 2008). This concept is proposed for the binding mechanism of DC-SIGN, which forms a tetramer on the surface of DCs and thereby increases affinity for its ligands mannan and gp120 (Mitchell et al., 2001; Bernhard et al., 2004). Additionally, DC-SIGN becomes organized in nanoscale clusters within lipid rafts, which further supports ligand binding and internalization by DCs (Cambi et al., 2004; Neumann et al., 2008). Finally, it was suggested that for the interaction between highly mannosylated structures and DC-SIGN additional secondary binding sites on the CRD are present that further increase the binding affinity of multivalent epitopes (Mitchell et al., 2001). Knowledge of binding and activation requirements have been exploited to target anti-cancer vaccines to DC-SIGN (Li R. E. et al., 2019). Similar targeting strategies could be applied to other CLRs, which may provide the opportunity to target different APC subsets, such as LCs (Porkolab et al., 2018; Neuhaus et al., 2019; Wamhoff et al., 2019).

## Interaction between CLRs and other PRR Family Members

Bacteria express a specific combination of PRR ligands. Complementary, PRRs have a specific distribution pattern among different APC subsets. Consequently, engagement of the same CLR can have a different immunological outcome as a result of different co-stimulation in that particular cell type. For example, engagement of MGL by *S. aureus* ST395 enhances IL-6 and IL-12 production by human moDCs (Mnich et al., 2019), whereas engagement of MGL by *C. jejuni* glycosylated proteins dampens cytokine production by the same cell type (van Sorge et al., 2009). Since it is known that MGL crosslinking does not induce cellular activation (van Vliet et al., 2013; Heger et al., 2018), these contrasting outcomes may result from differential TLR engagement for *S. aureus* (activating predominantly TLR2; Takeuchi et al., 2000) and *C. jejuni* (activating predominantly TLR4; Rathinam et al., 2009). A similar situation of differential co-stimulation may contribute to the different outcomes of langerin targeting by *S. aureus* and *Y. pestis*. In this example, langerin-mediated *S. aureus* recognition induces production of Th1/Th17 cytokines, which is generally accepted to promote *S. aureus* clearance (van Dalen et al., 2019a), whereas *Y. pestis* exploits langerin to gain entry into LCs to facilitate dissemination to the lymph nodes (Yang et al., 2015). The exact molecular mechanism underlying these differential outcomes of langerin engagement by these two pathogens is currently not known. It is however intriguing to speculate that langerin targeting modulates intracellular signaling of co-triggered TLRs or possibly alters intracellular routing resulting in different antigen presentation. In the LC context, it is important to realize that these cells appear to express a restricted repertoire of TLRs compared to DCs, with consensus on absence of TLR4, TLR8, and TLR9 (Flacher et al., 2006; van der Aar et al., 2007). Consequently, LCs show no or little response to classical bacterial PAMPs such as LPS (Flacher et al., 2006; van der Aar et al., 2007).

For some CLRs, modulation of signaling pathways from other PRRs is well-characterized. For example, the intracellular signaling pathways of DC-SIGN and TLR4 converge in response to fucose-containing LPS from *H. pylori*, which skews immune responses from Th1 to Th2 (Bergman et al., 2004; Gringhuis et al., 2014). The molecular details for Th2 skewing have been identified and result from activation of the atypical NF-kB family member Bcl-3 by DC-SIGN, which represses TLR-induced proinflammatory cytokine expression (Gringhuis et al., 2014). This opens up the possibility for therapeutic intervention; addition of anti-DC-SIGN blocking antibodies allow TLR activation to proceed normally, switching T cell responses toward Th1 (Gringhuis et al., 2014), which is thought to enhance pathogen clearance. Another example is the CLR Dectin-2, which interacts with TLR4 signaling after mannosylated LPS (man-LPS) stimulation (Wittmann et al., 2016). In contrast to BM-DCs from wild-type mice, BM-DCs from Dectin-2 and TLR4 knockout mice are unable to enhance IL-10 and TNFα production after stimulation with Man-LPS, suggesting synergy between these two PRRs (Wittmann et al., 2016). These examples illustrate the importance of studying CLR responses in the context of intact bacteria vs. purified components and also in the relevant APC subset to most closely resemble the complex biology of bacteria-APC interaction.

## Tools and Challenges in Screening for New Interactions

The described examples of CLR interaction with bacterial pathogens at different barrier sites highlight the importance of this PRR subfamily for pathogen detection and the instruction of appropriate downstream immune responses. As such, it is critical to identify new interactions to improve our understanding of immune tolerance, host defense and disease pathogenesis and apply this knowledge toward the development of new treatment and vaccines. Screening for new interactions between bacterial glycans and CLRs is challenging for multiple reasons. From a biological perspective, we are faced with an overwhelming diversity of glycans that are produced by bacterial species. For example, our gut microbiome contains an estimated 10^13^ − 10^14^ bacterial cells (Sender et al., 2016a,b), which produce a plethora of carbohydrate-active enzymes (CAZymes; Cantarel et al., 2009). Unfortunately, it is currently still challenging to predict glycan structures based on genome sequences. In addition, bacterial glycan expression is further regulated by specific environmental conditions in the gut. Interaction with neighboring bacteria may also affect the glycan composition, for example through secretion of glycan-hydrolyzing enzymes that modify (surface-associated) glycans from competitor bacteria. Screening for new interactions among this large diversity of glycans with different CLRs and elucidating their function requires a well-developed tool box. We will discuss developed tools below, as well as the advantages and disadvantages for each strategy.

CLRs can be recombinantly expressed as soluble extracellular domains, which facilitates screening of a diverse range of bacteria. Commercially available recombinant CLRs, both human and mouse, are often expressed as extracellular domains linked to a tag, such as IgG Fc (Hsu and Mahal, 2009; Maglinao et al., 2014; Mayer et al., 2018). Due to the physical nature of IgG Fc, the soluble CLR-fusion proteins are expressed as dimers, which increases avidity of the CLR-glycan interaction compared to single soluble domains. However, the dimeric presentation does not reflect the natural arrangement of several CLRs, such as MGL, langerin and DC-SIGN, as trimers or tetramers on the cell surface (Feinberg et al., 2005, 2010; Jegouzo et al., 2013). In addition, Fc-binding proteins are expressed by several bacterial species (Sidorin and Solov'eva, 2011; Nordenfelt and Bjorck, 2013), such as *S. aureus* and *S. pyogenes*, and likely by other species, which further challenges screening for new interactions with these specific human pathogens due to high non-specific background binding. The use of non-Fc-based tags (such as Strep-tag II; Hanske et al., 2016), orientation of the Fc-tagged constructs on arrays or on protein A/G-coated ELISA plates are possible solutions to prevent non-specific Fc-mediated binding (Chen et al., 2008). Alternatively, lectins are spotted on an array, which can then be exposed to bacterial components or whole bacterial cells (Hsu et al., 2006). This platform is able to produce a specific bacterial fingerprint and can assess dynamic changes to the bacterial glycan coat (Hsu et al., 2006). The mentioned platform used well-defined plant lectins to probe the bacterial glycan profile. A similar platform consisting of mammalian CLRs would be a very valuable tool to identify new CLR-bacteria interactions. Finally, CLRs can be ectopically expressed on cell lines, potentially as GFP-reporter constructs (Imai et al., 2018). This approach conserves the natural multimerization state of the receptor, but usually provides only a limited window in which specific interactions can be observed. Furthermore, GFP-reporter cell lines will only identify activating ligands, whereas CLR antagonists remain undetected. Additionally, since not all CLRs contain intrinsic signaling capacity, they need to be expressed as chimeric constructs for example with the CD3ζ chain, which may alter their properties in unknown ways (Imai et al., 2018).

With the aforementioned approaches, specific CLRs can be screened for interactions using intact bacteria. Alternatively, CLR constructs are used to screen glycan arrays (Rillahan and Paulson, 2011; Geissner et al., 2019), which display isolated bacterial glycans. This approach may benefit from the multivalent glycan display, which increases avidity of the interaction, allowing for identification of low affinity interactions. However, isolation of glycans from bacteria can be challenging due to the presence of labile groups or modifications that are lost during sample preparation (Lewis et al., 2004; Edgar et al., 2019). This has sparked the development of synthetic carbohydrate chemistry (Adamo, 2017; van der Es et al., 2017; Zhang and Ye, 2018), which allows the generation of libraries consisting of stable and well-defined glycan structures. In addition, these defined structures enable structure-function studies and crystallography (Gerlach et al., 2018). Crystallography with synthetic glycans is a useful approach once a CLR-glycan interaction is established but due to the vast number of glycosylated bacterial structures it does not allow for a comprehensive approach to screen for new glycan interactions across the entire bacterial kingdom. Complementary use of all mentioned tools is therefore essential to identify new interactions between bacteria and host CLR.

## Model Systems to Assess Functional Consequences of CLR Interaction

The above-mentioned studies have provided insight into the functional consequences of CLR-bacteria interactions. However, experimental work with APCs is not without technical and logistical challenges. First and foremost, the transcriptional profiles of APCs from the same subset can differ significantly depending on the tissue where they are isolated from, as cells receive specific signals from their surrounding microenvironment (Lundberg et al., 2013). Consequently, APCs should ideally be isolated from the tissue of interest and be related to the infection route of the bacterium. However, isolation of primary APCs is not feasible for every tissue or for every laboratory due to limited availability or accessibility to human tissues. Even if the relevant tissues are obtained, it is challenging to isolate a suitable number of immature cells for experiments, due to limited cell numbers and (partial) induction of APC maturation by the isolation procedure (Botting et al., 2017). Therefore, APCs differentiated from blood monocytes or CD34+ cord blood cells by a specific cocktail of cytokines, have become widely-used models to study APC- and CLR-induced responses. Using these differentiation strategies, APCs with an immature phenotype can be obtained and differentiated into mature APCs in a controlled manner. In addition to human primary cells, the use of cell lines that can be differentiated in distinct APCs subsets, such as the myeloid CD34+ MUTZ3 cell line (Masterson et al., 2002; Santegoets et al., 2006), have yielded more insight into CLR-mediated interaction and responses (de Jong et al., 2010a; van Dalen et al., 2019a). The advantages of this cell line-based approach are the absence of donor-to-donor variability, independence on donor-derived material and amenability of these cells to CRISPR/Cas9 genetic manipulation, which makes these cells suitable to address the role of specific molecules in immunological interactions. Another possibility to generate APCs is through induced pluripotent stem cells (iPSC) derived from fibroblasts, which offers an unlimited cell source from the same individuals and the possibility to generate patient-specific APCs with genetic defects (Choi et al., 2009, 2011; Yanagimachi et al., 2013; Ackermann et al., 2015). Although these approaches are understandably widely applied for ease of use and quantity of cells, transcriptomics studies have made clear that all *in vitro* differentiated cell and cell lines are distinct from primary cells (Lundberg et al., 2001; Harman et al., 2013). For example, MUTZ3-derived LCs express several TLRs that are not expressed by primary LCs (Lundberg et al., 2013). Therefore, data obtained with differentiated primary cells, cell lines and iPSC should be interpreted with care, and experiments should be reproduced in more physiologically relevant primary cells, *ex vivo* tissue models or suitable *in vivo* models.

Fortunately, in recent years, several *ex vivo* and 3D tissue models have been developed. Human skin explants have been used to study the interaction between different cell populations and common skin pathogens, including *S. aureus* (Schaudinn et al., 2017; Olaniyi et al., 2018). This approach is currently the most relevant model to study the interaction of CLRs with bacteria in their natural environment, since the explanted tissue contains all relevant cell populations. Since not all laboratories have access to human skin, an alternative approach is the use of designed skin equivalents consisting of either epidermis alone or together with underlying dermis. Progress is made to repopulate these organotypic skin models with specific skin-resident immune cells (Pupovac et al., 2018), for example CD34+ progenitor cells that differentiate into LCs when co-seeded with human keratinocytes (Regnier et al., 1998). DCs can also be repopulated in the skin equivalent model by introducing human moDCs in between layers of keratinocytes and fibroblasts (Chau et al., 2013). Not only for skin, but also for lung tissue, 3D tissue models have been generated to study molecular pathogenesis of bacterial infections, such as *S. aureus* toxin-mediated lung pathology relevant for pneumonia (Mairpady Shambat et al., 2015). Also here it was possible to repopulate the model with functional human moDCs (Mizoguchi et al., 2017).

The development of organoid technology has brought about a surge in the use of 3D models, also for studies in the area of infectious diseases (Schutgens and Clevers, 2020). There are now a wide variety of tissues and organs that can be grown from human pluripotent stem cells (hPSCs) with a specific cocktail of growth factors including lung, gut, stomach and even brain organoids (Clevers, 2016). Since organoids consist of all cellular components of the representative organ, they provide opportunities to study bacteria-host interactions in a more physiologically-relevant setting as compared to cell lines. For example, gastric organoids have been used to study host responses to *H. pylori* (Bartfeld et al., 2015). Despite many advantages, organoids have limitations such as the absence of blood vessels and immune cells, which are crucial aspects of host defense against infections. The development of immune-competent organoids models (Bar-Ephraim et al., 2020) will provide an opportunity to study interactions between CLR and bacteria in the fully differentiated context of different human tissues. Overall, all immunocompetent 3D tissue models can be useful tools to gain insight into bacterial-cell interactions and the influence of the specific environmental context on the functional outcomes of this interactions, i.e., influence of cell-cell communication.

## Conclusion and Future Outlook

From their first discovery, CLRs have been studied mainly for their role in anti-fungal and anti-viral immunity, with research on CLR-bacterial interactions lagging behind. Our overview of bacterial-CLR interactions clearly highlights the importance of specific CLRs in anti-bacterial immunity, but also provides examples of pathogens such as *Y. pestis* that exploit CLR interaction to enhance virulence or survival. Studies have generally focused on the role of CLRs in the detection of bacterial pathogens. However, APCs also actively sample the environment across intact tissue barriers, suggesting an important role in immune homeostasis that has yet to be elucidated. A major challenge in the identification of new interactions of CLRs with bacterial species in the microbiome is the vast diversity of bacterial glycan structures, in combination with technical challenges that have to be overcome to close this knowledge gap. In this review, we have discussed advantages and disadvantages of currently available approaches. Applications of CLR-bacterial interactions include the development of targeting agents for vaccine delivery to specific CLRs on APC subsets, that can help boost an effective adaptive immune and memory response. In this regard, the infectious diseases field should take advantage of the progress in the area of cancer vaccinology, where studies have shown the benefit of *in vivo* targeting of cancer antigens to APCs to enhance anti-tumor immunity (Hossain and Wall, 2019). Overall, the knowledge gained from studies on bacterial-CLR interactions could therefore not only shed light on the role in immune defense or pathogenicity but also be highly relevant to the translation of vaccine applications.

## Author Contributions

MM, RD, and NS wrote, revised, and approved the manuscript. MM prepared the figure and table. All authors contributed to the article and approved the submitted version.

## Conflict of Interest

MM is a Ph.D. fellow who is enrolled in the Infection and Immunity Ph.D. program, part of the Graduate School of Life Sciences at Utrecht University, and participates in a postgraduate studentship program at GSK. The remaining authors declare that the research was conducted in the absence of any commercial or financial relationships that could be construed as a potential conflict of interest.
